# Summer Knitting

**Published:** 1917-06-30

**Authors:** John Penoyre

**Affiliations:** 8 King's Bench Walk, Inner Temple, E.C. 4.


*To the Editor of* THE HOSPITAL.

### Summer Knitting.

*To the Editor of* THE HOSPITAL.

SIR,—Can you find me room to express deep gratitude to friends of sweaters all over the country who, I hear, are getting together a wonderful supply of these and other comforts for the autumn? Even a man knows that heavy knitting cannot be congenial this weather. Never mind, the results will be, to the wearers in winter. Anything knitted for me should be held over till the third week in July, when I will be careful to give the address to which the sacks and parcels should be sent. I have long outgrown the space here.

I have plenty of printed patterns of easily knitted comforts left, if ladies will kindly write for them.—Yours faithfully, JOHN PENOYRE.

8 King's Bench Walk, Inner Temple, E.C. 4.,

June 26, 1917.

